# A novel mutation in the NADH dehydrogenase (ubiquinone) 1 alpha subcomplex 4 (*Ndufa4*) gene links mitochondrial dysfunction to the development of diabetes in a rodent model

**DOI:** 10.1242/dmm.036699

**Published:** 2018-11-20

**Authors:** Chana Yagil, Ronen Varadi-Levi, Yoram Yagil

**Affiliations:** 1 Laboratory for Molecular Medicine and Israeli Rat Genome Center, Barzilai University Medical Center, Ashkelon 7830604, Israel; 2 Faculty of Health Sciences, Ben-Gurion University of the Negev, Beer Sheba 8410501, Israel

**Keywords:** Cohen diabetic rat, Diabetogenic diet, Genetics, Mutation, Mitochondria

## Abstract

The mechanisms underlying diabetes remain unresolved. The Cohen diabetic rat represents a model of diet-induced diabetes, in which the disease is induced after exposure to a diabetogenic diet (DD) in the diabetes-sensitive (CDs/y) but not in the -resistant (CDr/y) strain. Diet imposes a metabolic strain that leads to diabetes in the appropriate genetic background. We previously identified, through whole-genome linkage analysis, a diabetes-related quantitative trait locus on rat chromosome 4 (RNO4), which incorporates NADH dehydrogenase (ubiquinone) 1 alpha subcomplex 4 (*Ndufa4*), a nuclear gene that affects mitochondrial function. Here, we sequenced the gene and found a major deletion in CDs/y that leads to lack of expression of the NDUFA4 protein that has been reported to be involved in the activities of mitochondrial complexes I and IV. In the absence of NDUFA4 in the diabetic CDs/y on DD, complex I activity is reduced in comparison to that in nondiabetic CDs/y on regular diet and CDr/y on either diet; complex IV activity is reduced in both strains provided DD, and thus as a result of diet and unrelated to the gene mutation. ATP fails to increase in diabetic CDs/y in response to DD, in comparison to nondiabetic CDr/y on DD. Plasma malondialdehyde levels are elevated in CDs/y on DD, whereas SOD1 and SOD2 levels fail to increase, indicating increased oxidative stress and inability of the pancreas to generate an appropriate antioxidative stress response. These findings suggest that the *Ndufa4* mutation in CDs/y on DD is directly associated with mitochondrial dysfunction, which we attribute to the lack of expression of NDUFA4 and to diet, and which prevents the anticipated increase in ATP production. The resulting enhanced oxidative stress impairs the ability of the pancreas to secrete insulin, leading to the development of diabetes. This is the first demonstration of an inherited mutation in a nuclear gene that adversely affects mitochondrial function and promotes diet-induced diabetes.

## Abstract (precis)

**Summary:** Here, we report, for the first time, a major inherited mutation in a diabetes-prone animal model that adversely affects mitochondrial function and leads, through oxidative stress, to the development of diet-induced diabetes.

## INTRODUCTION

The pathophysiology of diabetes remains incompletely understood. Multiple studies over the past decades in humans and in animal models have indirectly implicated the mitochondria in the pathogenesis of diabetes and its complications (Chow et al., 2017; Lowell and Shulman, 2005). We currently provide novel evidence of an inherited mutation in a candidate gene that links mitochondrial dysfunction to the development of diet-induced diabetes.

The Cohen diabetic rat is an experimental model reminiscent of type 2 diabetes in humans. The model consists of two substrains: Cohen diabetic-sensitive (CDs/y) and Cohen diabetic-resistant (CDr/y) rats (Weksler-Zangen et al., 2001). Both substrains exhibit a normal metabolic profile while fed a regular diet (RD), but when provided a custom-prepared diabetogenic diet (DD), CDs/y invariably develop diabetes, whereas CDr/y do not (Weksler-Zangen et al., 2001). Genetic linkage studies in this model have led us to uncover a genomic locus on rat chromosome 4 (RNO4) that incorporates in the rat – and in syntenic regions in mouse and human – several candidate genes, including NADH dehydrogenase (ubiquinone) 1 alpha (*Ndufa4*) (Barkalifa et al., 2010). *Ndufa4*, a nuclear gene, encodes the NDUFA4 protein that makes up at least one of the subunits of the mammalian oxidative phosphorylation (OXPHOS) complexes, and which exerts its effect within the mitochondria (Mishmar et al., 2006).

In the current study, we investigated – in CDs/y and CDr/y – the *Ndufa4* gene sequence, the gene transcript, its expression at the protein level and mitochondrial function, with a focus on complexes I and IV, in which this gene has been previously implicated (Balsa et al., 2012; Garbian et al., 2010; Hayashi et al., 2009; Mishmar et al., 2006; Pitceathly et al., 2013; Sinkler et al., 2017). We present the results, which demonstrate in CDs/y a novel mutation within the *Ndufa4* gene that accounts for the lack of expression of the intact NDUFA4 protein, which, in the appropriate environment, leads to impaired mitochondrial function, increased oxidative stress and the development of diet-induced diabetes mellitus.

## RESULTS

### Sequencing of the *Ndufa4* gene

#### Exon sequencing

**Fig. 1.**
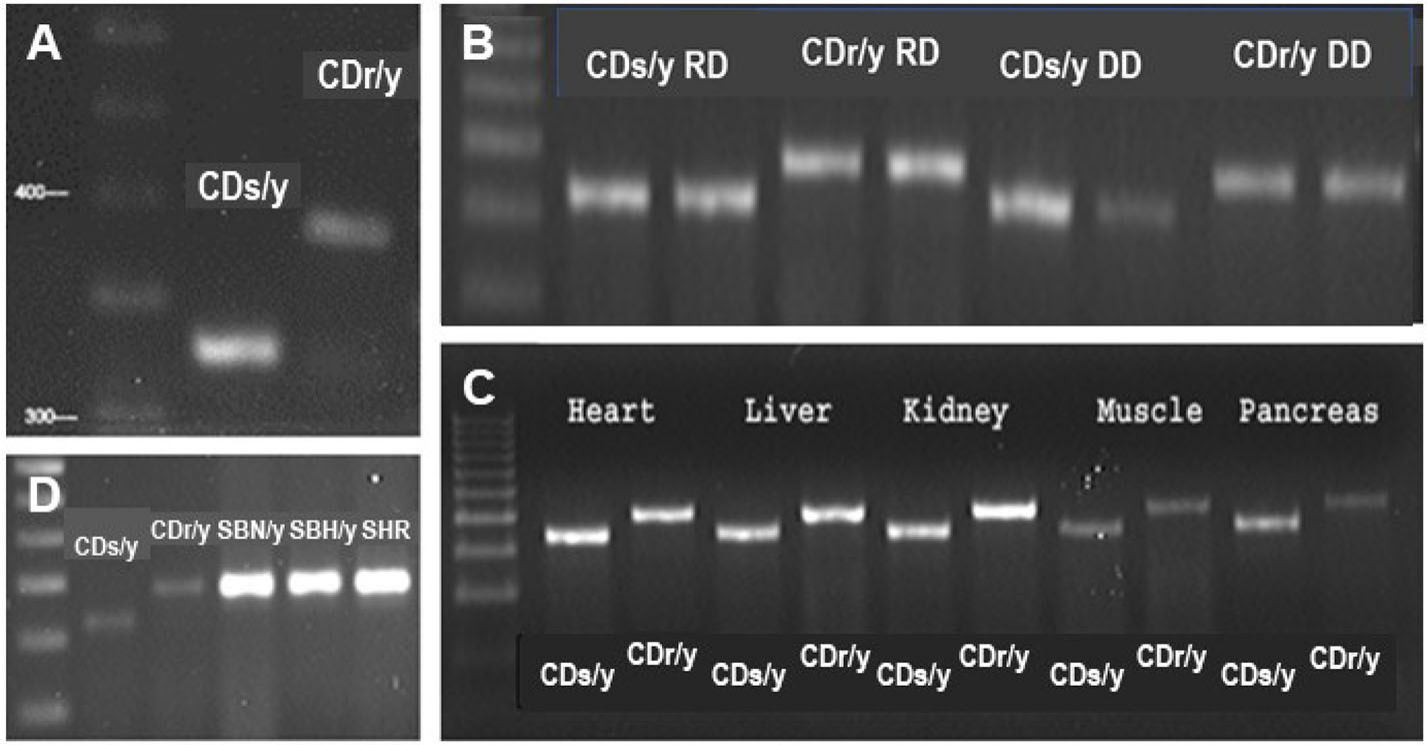
**PCR products of *Ndufa4* transcript cDNA amplification.** (A-D) *Ndufa4* cDNA was amplified in pancreatic extracts from CDs/y and CDr/y provided RD (A), pancreatic extracts from CDs/y and CDr/y provided RD or DD (B), extracts from other organs in CDs/y and CDr/y on RD (C) and pancreatic extracts from other unrelated rat strains (D).

**Fig. 2.**
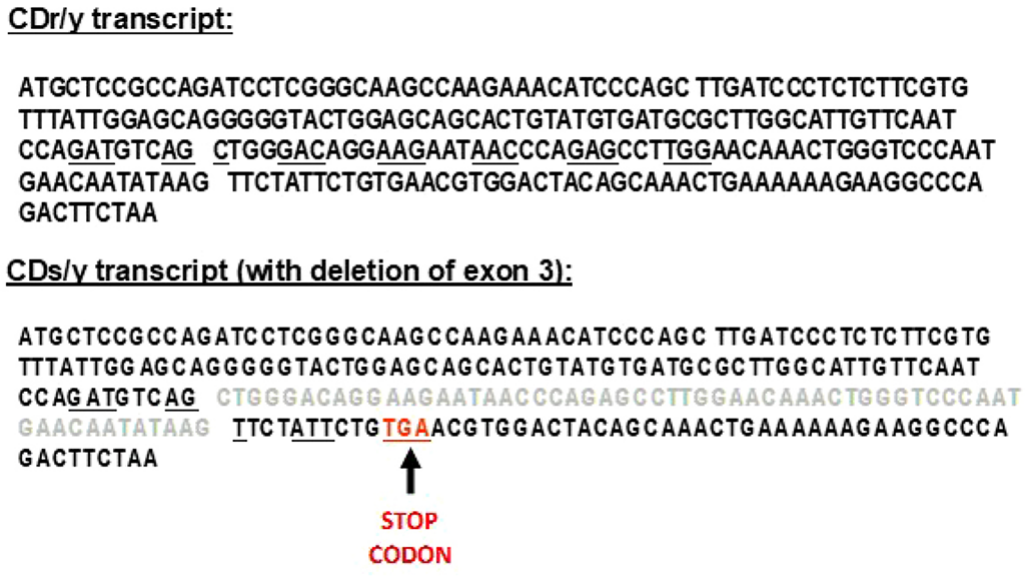
***Ndufa4* gene transcript in CDs/y and CDr/y.** Intact *Ndufa4* gene transcript in CDr/y (top) and incomplete gene transcript in CDs/y with deletion of exon 3 (in light gray) (bottom), causing a frameshift mutation and leading to the formation of a TGA stop codon (red). Underlined adjacent nucleotides represent codons defining amino acids for protein synthesis.

We amplified *Ndufa4* complementary DNA (cDNA) from pancreatic extracts of the two strains and found a difference in size of the PCR product: CDs/y 330 bp versus CDr/y 390 bp (Fig. 1A), regardless of whether the animals were on DD or RD, or expressed the diabetic phenotype or not (Fig. 1B). We found the same shorter PCR product size in CDs/y in the heart, liver, kidney and muscle (Fig. 1C), but not in other nondiabetic rat strains, including Sabra hypertension-prone (SBH/y), Sabra hypertension-resistant (SBN/y) and spontaneously hypertensive rat (SHR) (Fig. 1D). We sequenced the gene and found that the short PCR product in CDs/y was due to skipping of exon 3, whereas exons 1, 2 and 4 were identical in CDs/y and CDr/y (Fig. 2). The size difference in the PCR products between the two strains was accounted for in full by the 61 bp size of *Ndufa4* exon 3 in the rat.

#### Genomic sequencing

In CDr/y, we successfully amplified the entire *Ndufa4* gene, consisting of four exons and three introns. In CDs/y, we were able to amplify only exons 1, 2 and 4, and introns 1 and 2, but were unsuccessful in amplifying exon 3 or intron 3. To investigate further the lack of amplification of exon 3 and intron 3 in CDs/y, we used Phusion High-Fidelity DNA Polymerase to amplify a big PCR product from the middle of intron 1 and up to 110 bp downstream from exon 4, thereby covering exon 3 and intron 3. In CDr/y, the PCR product was ∼2800 bp larger than in CDs/y. We sequenced the big PCR product and confirmed its belonging to the published *Ndufa4* gene sequence. We then designed primers for small PCR products to cover the gene components demarcated by the large PCR products. We successfully amplified several small PCR products in CDr/y but not in CDs/y. Using ‘gene walking’ and primers designed to regions proximal and distal to the unamplified genomic segment, we confirmed the size difference between CDs/y and CDr/y. The amplified PCR product from CDs/y revealed that the gene sequence jumped from the end of intron 2 (bp 1859 – the last part of the 5′ spliceosome of exon 3) directly to the middle of intron 3, thereby skipping 2793 bp.

### Expression of the NDUFA4 protein

**Fig. 3.**
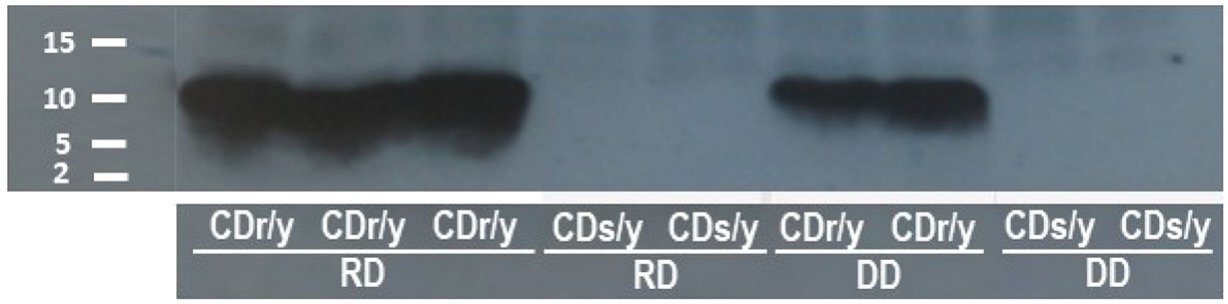
**Expression of the NDUFA4 protein by western blotting.** The protein is expressed in CDr/y but not in CDs/y; the level of expression is markedly greater in CDr/y fed RD than in CDr/y provided DD.

We identified, by western blotting, the NDUFA4 protein in CDr/y fed RD and those fed DD, but were unable to demonstrate the protein in CDs/y on either diet (Fig. 3). We confirmed these results in whole-cell homogenates, in membranous-enriched samples and in soup protein extracted from the pancreas, liver, kidney and muscle. In CDr/y, the level of expression varied with diet and, in animals fed RD, was double to triple the amount found in animals on DD (Fig. 3).

### Localization of NDUFA4 within the electron chain complex

**Fig. 4.**
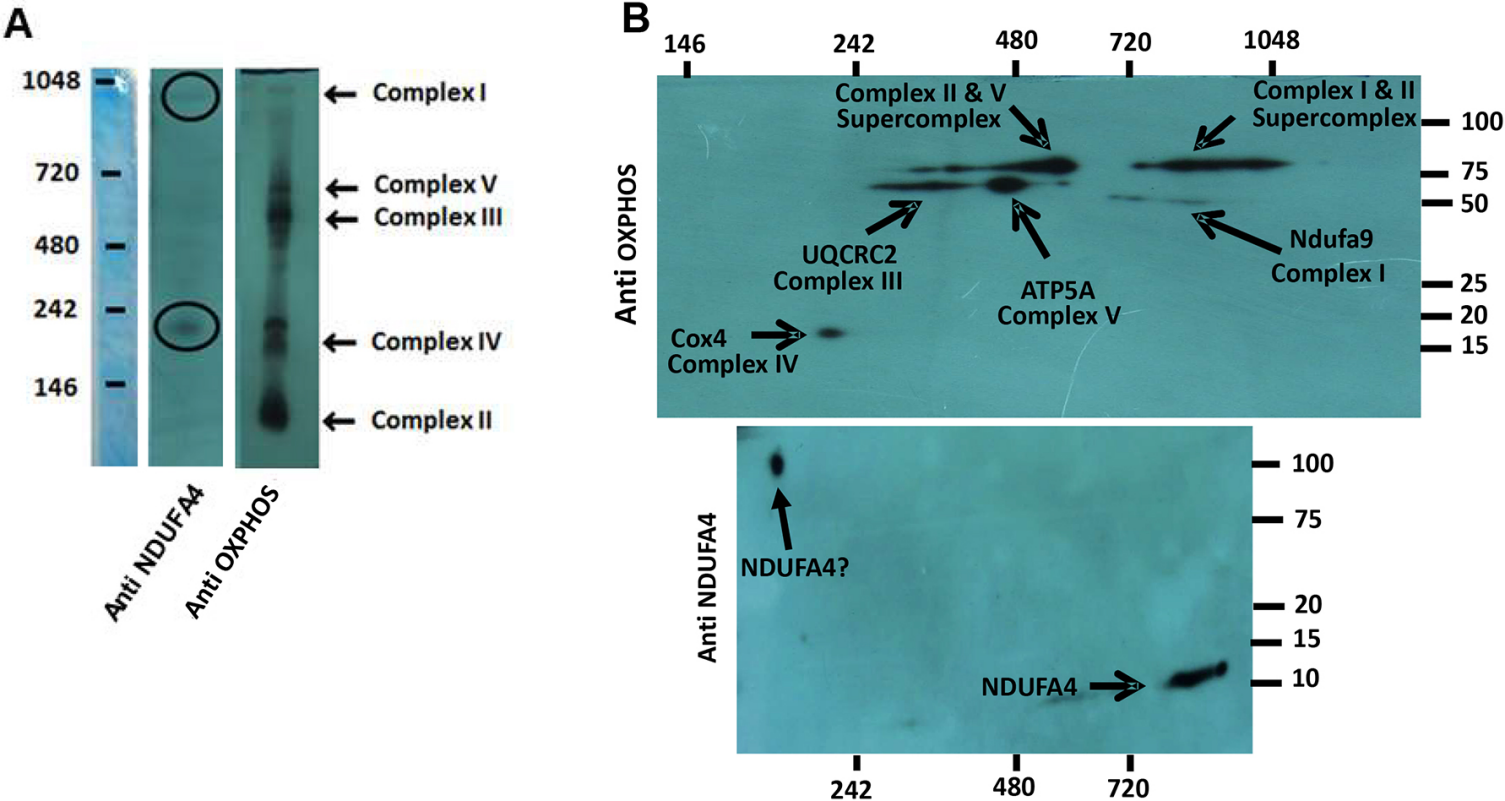
**Localization of NDUFA4 within the mitochondrial electron chain complexes.** (A,B) Localization of NDUFA4 by denaturing 1D western blot within the mitochondrial electron chain complexes I and IV (A; consisting of a composite of a ladder and two separate blots on the same membrane, one with the anti-NDUFA4 antibody and the other with OXPHOS) and by 2D blue native gels, with a molecular mass of ∼10 kDa for NDUFA4 in complex I and a molecular mass of 75-100 kDa in complex IV (B; also consisting of a composite of two separate blots on the same membrane, one with the anti-NDUFA4 antibody and the other with OXPHOS).

The 1D blue native gel (Fig. 4A) placed the NDUFA4 protein at ∼900 kDa, corresponding to the complex I region, and at ∼200 kDa, in proximity to complex IV. The 2D blue native gel (Fig. 4B) confirmed the location of the NDUFA4 protein molecular mass ∼10 kDa within complex I, but identified an additional band with molecular mass between 75 kDa and 100 kDa in the vicinity of complex IV. The large size of the second molecule, well above that of NDUFA4, suggests either nonspecific binding at that site or that NDUFA4 is bound to a subcomplex of complex I that has been dissociated in the denaturing conditions. We found the same pattern in CDr/y under RD or DD, and also in tissue extracted from the liver and kidney (data not shown).

### OXPHOS abundance

**Fig. 5.**
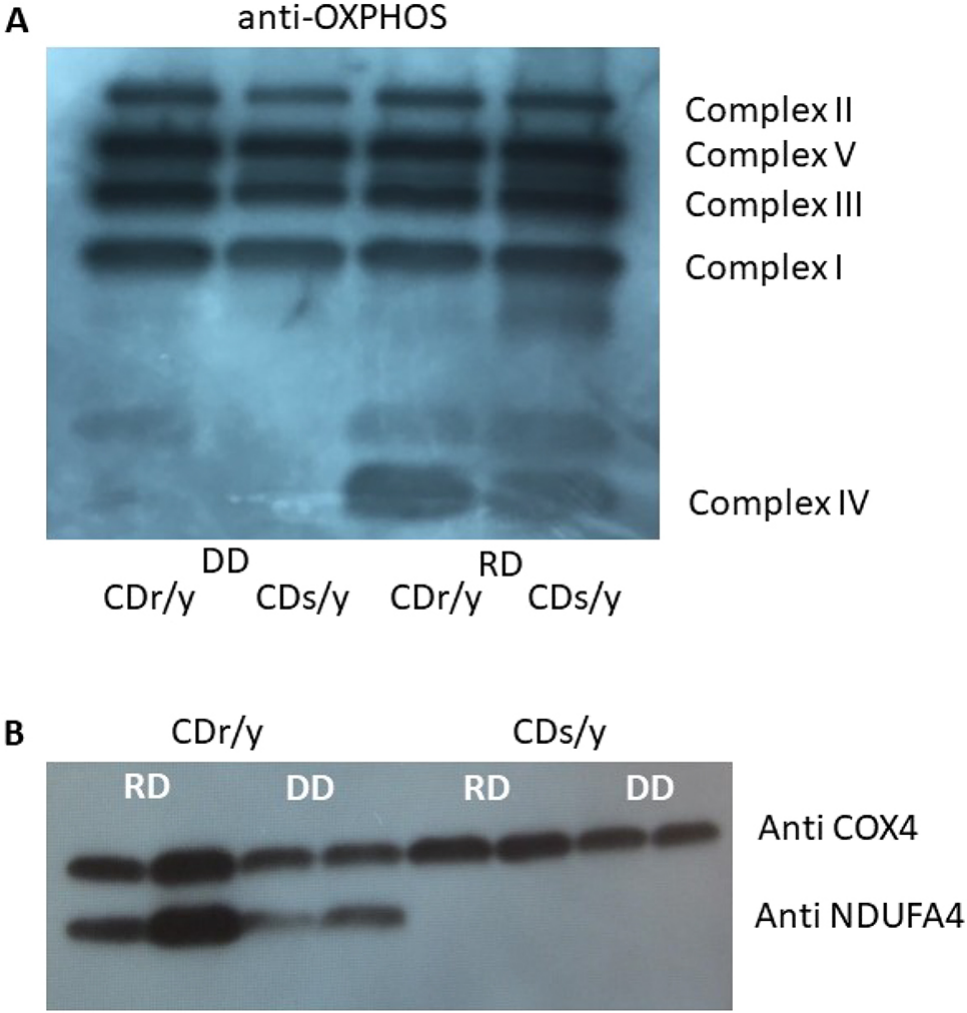
**Mitochondrial complex abundance.** (A) Anti-OXPHOS cocktail showing mitochondrial complex abundance in CDs/y and CDr/y provided RD or DD; there is similar expression of proteins in complexes I, II, III and V in CDs/y and CDr/y provided RD or DD, but lower abundance of complex IV expression in both strains when provided DD versus RD. (B) This finding was confirmed with COX4 (also known as COX4I1), a component of complex IV, for which expression is lower when fed DD versus RD, irrespective of strain.

We found similar abundance of the proteins composing each of the complexes I, II, III and V, with no difference between CDr/y and CDs/y, whether on RD or DD (Fig. 5A). Within complex IV, however, we found lower OXPHOS protein abundance in the animals on DD compared with those on RD, irrespective of strain. This finding was confirmed by studying specifically COX4 expression (a component of complex IV) that was reduced in CDs/y and CDr/y provided DD, compared with those provided RD (Fig. 5B). In parallel, NDUFA4 abundance was reduced in CDr/y on DD compared with those on RD, whereas protein expression was totally lacking in CDs/y (Fig. 5B).

### Mitochondrial function

#### Complex I activity

**Fig. 6.**
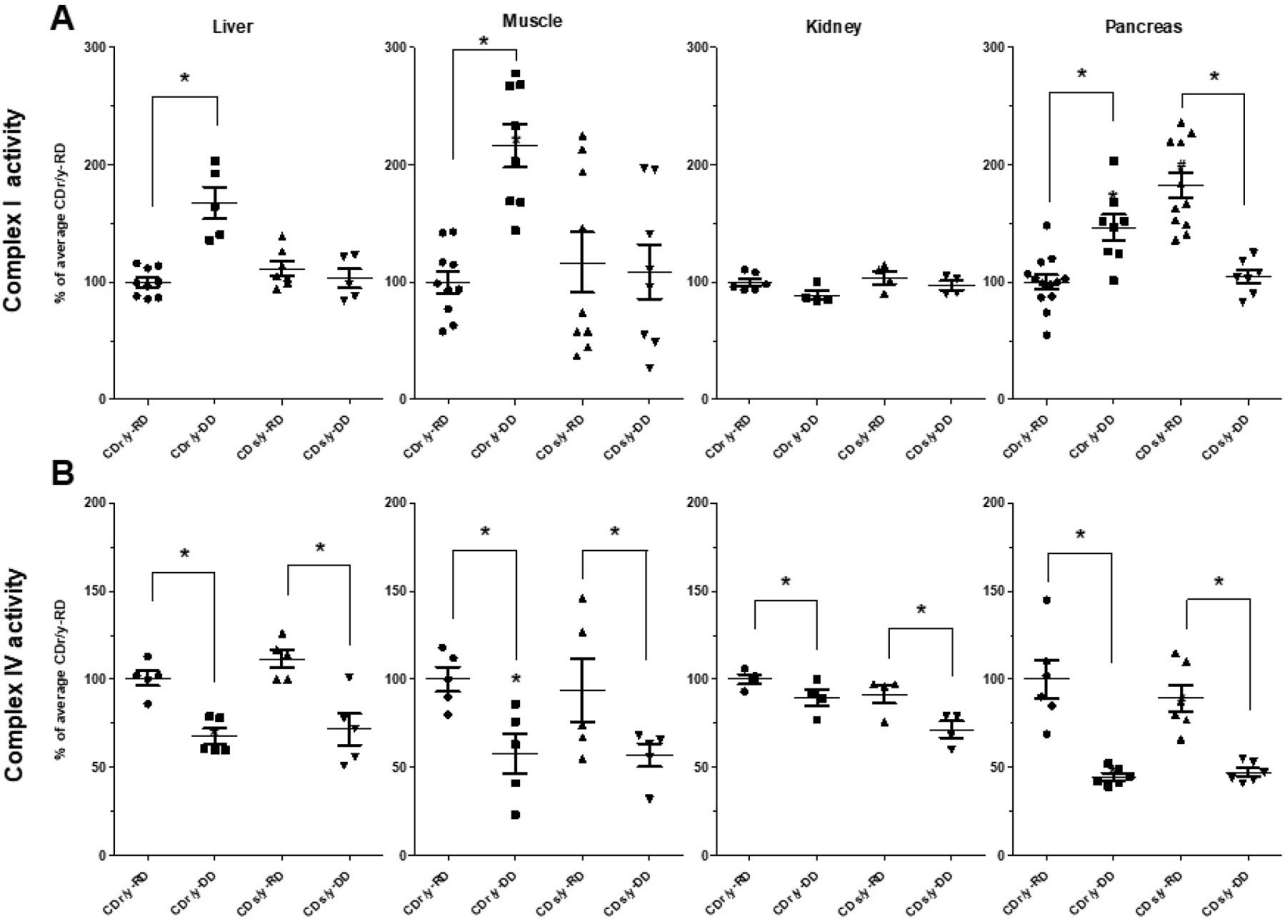
**Activities of complexes** **I and IV in the liver, muscle, kidney and pancreas of CDs/y and CDr/y on RD or DD.** (A) Complex I activity. (B) Complex IV activity. *P<0.01 by ANOVA and post hoc LSD testing; DD versus RD within each strain; data are mean±s.e.m.

To compare activity between CDs/y and CDr/y under RD and DD, we focused our studies on the liver, muscle, kidneys and pancreas (Fig. 6A). In the liver, muscle and kidney, complex I activity was similar in CDs/y and CDr/y on RD; in those on DD, however, the activity was higher by 60-100% in the liver and muscle of CDr/y, but did not change in CDs/y, whereas in the kidney, such changes did not occur. In the pancreas, complex I activity showed a different pattern of response to diet than in the other organs: While on RD, complex I activity was ∼80% higher in CDs/y than in CDr/y, whereas on DD, the activity in CDs/y was ∼40% lower than in CDr/y

#### Complex IV activity

Complex IV activity in the liver, muscle, kidney and pancreas was similar in CDr/y and CDs/y provided RD (Fig. 6B). When provided DD, complex IV activity declined significantly in both CDr/y and CDs/y.

#### Adenosine triphosphate (ATP) production

**Fig. 7.**
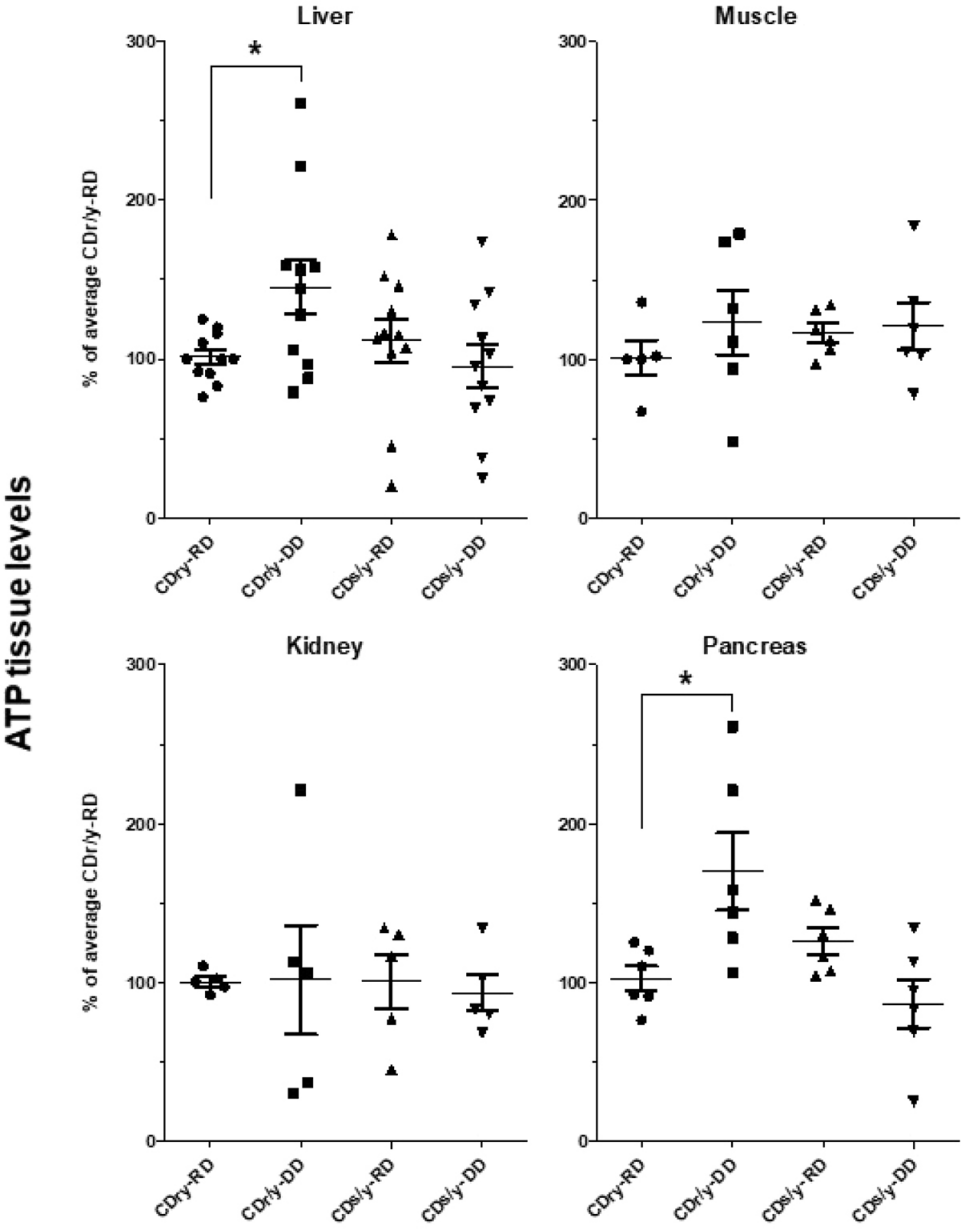
**Tissue ATP levels measured in the liver, muscle, kidney and pancreas of CDs/y and CDr/y on RD or DD.** *P<0.01 by ANOVA and post hoc LSD testing; DD versus RD within each strain; data are mean±s.e.m.

In animals provided RD, ATP levels in the liver, muscle, kidney and pancreas were not different between CDr/y and CDs/y (Fig. 7). When provided DD, ATP levels increased significantly in CDr/y in the liver and in the pancreas, but only marginally in muscle and not in the kidney. In CDs/y fed DD, ATP levels failed to increase within muscle, kidney, liver and pancreas.

### Oxidative stress

#### Malondialdehyde (MDA) serum levels

**Fig. 8.**
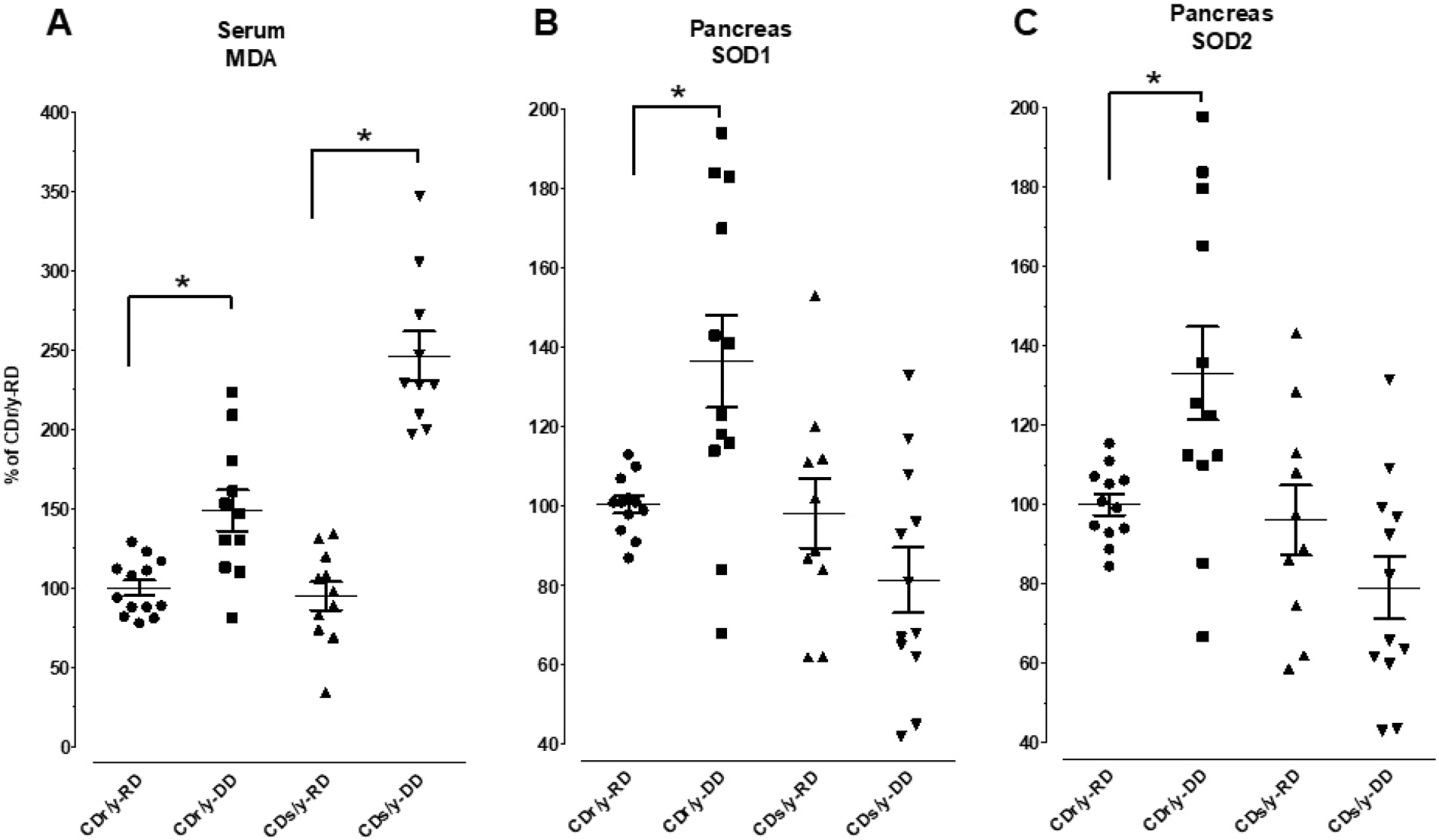
**Serum MDA, and pancreatic SOD1 and SOD2 levels in CDs/y and CDr/y on RD or DD.** (A) Serum MDA. (B) Pancreatic SOD1. (C) Pancreatic SOD2. *P<0.01 by ANOVA and post hoc LSD testing; DD versus RD within each strain; data are mean±s.e.m.

MDA serum levels were significantly elevated in CDs/y on DD compared with all other groups (Fig. 8A). Interestingly, serum MDA levels also rose significantly in CDr/y when fed DD, as compared with levels when fed RD, but the increment was less than half of that observed in CDs/y.

#### SOD1, SOD2

Pancreatic SOD1 and SOD2 levels were higher in nondiabetic CDr/y on DD compared with nondiabetic CDr/y provided RD (Fig. 8B,C). In CDs/y fed RD, SOD1 and SOD2 levels were not different from those in CDr/y provided RD. However, in contrast to CDr/y, in CDs/y fed DD, SOD1 and SOD2 protein levels failed to rise and even tended to decrease (Fig. 8B,C).

## DISCUSSION

The current in-depth exploration of *Ndufa4* as a mitochondrial functional candidate gene involved in the pathogenesis of diet-induced diabetes was driven by our previous localization of the gene by linkage analysis to a diabetes-related quantitative trait locus (QTL) on rat chromosome 4 (RNO4), and the differential expression of the gene between the diabetes-prone CDs/y and -resistant CDr/y strains (Barkalifa et al., 2010; Yagil et al., 2007, 2016). We now report on a novel mutation in the *Ndufa4* gene in CDs/y that is not present in CDr/y, and that consists of a large deletion, resulting in the skipping of exon 3 and part of intron 3, and creating a frameshift mutation and a stop codon (Fig. 2). We confirmed this deletion in the *Ndufa4* gene by next-generation sequencing and determined that this sequence variation was unique to CDs/y, among 25 other diabetic and nondiabetic rat strains (Yagil et al., 2016).

Based on the difference in gene structure between CDs/y and CDr/y, the predicted translation of *Ndufa4* leads to a 45-amino-acid peptide in CDs/y (up to the stop codon), instead of the intact 81-amino-acid protein in CDr/y. As a result, the intact NDUFA4 protein is expressed in CDr/y under RD or DD, but the *Ndufa4* gene mutation in CDs/y prevents altogether the expression of the intact NDUFA4 protein.

The NDUFA4 protein is a 9 kDa subunit that has NADH dehydrogenase and oxidoreductase activities and transfers electrons from NADH to the respiratory chain, the immediate electron acceptor thought to be ubiquinone. The protein has previously been identified as part of the 45 different subunits that compose the mammalian complex I of the respiratory mitochondrial respiratory chain (GeneCards, http://www.genecards.org/cgi-bin/carddisp.pl?gene=Ndufa4) (Garbian et al., 2010; Hayashi et al., 2009; Mishmar et al., 2006; Pitceathly et al., 2013). More recent data suggest that the NDUFA4 protein might be related to the mitochondrial cytochrome c oxidase (COX, complex IV) activity, as the terminal component of the mitochondrial chain that catalyzes the reduction of oxygen to water and that it is required for complex IV maintenance (GeneCards, UniProtKB/Swiss-Prot) (Balsa et al., 2012; Pitceathly et al., 2013). Irrespective of whether NDUFA4 locates to complex I or IV, we reasoned that the absence of its expression in CDs/y would carry functional implications and affect mitochondrial function that might be associated with the development of diabetes. We set out, therefore, to investigate mitochondrial function in CDs/y and CDr/y fed RD or DD.

Prior to studying the effect of the lack of expression of the NDUFA4 protein on mitochondrial function, we sought to determine whether the mutation in CDs/y affects the integrity of the components making up the mitochondrial respiratory chain. We demonstrated, through OXPHOS studies, that the size and abundance of the individual mitochondrial complexes I, II, III and V were unperturbed in CDs/y compared with CDr/y on either diet, attesting to their intactness in CDs/y, despite the *Ndufa4* mutation. We did observe reduced abundance of complex IV in both CDs/y and CDr/y fed DD, which we attributed to the effect of diet and not to the *Ndufa4* mutation or to the diabetic phenotype, which do not appear in CDr/y. We then attempted to clarify further the controversy whether NDUFA4 locates within the mitochondrial respiratory chain to complex I (Garbian et al., 2010; Hayashi et al., 2009; Mishmar et al., 2006; Pitceathly et al., 2013) or IV (Balsa et al., 2012; Pitceathly et al., 2013). Our findings indicate that, in the rat, NDUFA4 locates to complex I, as originally determined. On the other hand, we cannot rule out with certainty its location also within complex IV, even though in the latter complex, NDUFA4 appears with a molecular mass between 75 kDa and 100 kDa, well above the ∼9 kDa attributed to the protein, suggesting either nonspecific binding at that site or that NDUFA4 is bound to a subcomplex of complex I that has been dissociated in the denaturing conditions. Our findings thus reposition, with a great degree of confidence, NDUFA4 to complex I, but the possibility that NDUFA4 or a similar protein might also localize to complex IV remains open and requires additional investigation.

As we could not rule out the possibility that the two mitochondrial complexes might involve NDUFA4, we studied the activity of both complex I and IV activities in CDs/y (in which NDUFA4 is not expressed) and compared it with that in CDr/y (in which the intact protein is fully expressed). In the basal state, during which the animals are provided RD, complex I activity in CDs/y is not different from that in CDr/y in the liver, muscle and kidney, indicating that, in these organs, the *Ndufa4* mutation and the lack of expression of the NDUFA4 protein do not affect basal mitochondrial function. In the pancreas of CDs/y fed RD, however, complex I activity is unexpectedly elevated, compared with that in the pancreas of CDr/y provided RD, which could be interpreted as an organ-specific requirement for increased activity to compensate for the absence of the NDUFA4 subunit. In contrast to the basal state when animals are fed RD, when CDr/y are fed DD, the dietary diabetogenic stimulus, complex I activity increases significantly in the liver, muscle and pancreas, indicating an increase in mitochondrial activity, most likely in response to enhanced demand induced by the DD. Interestingly, complex I activity in the kidney does not increase, suggesting that the kidney, as opposed to the liver and muscle, may not actively participate in the regulation of energy metabolism. Contrary to what we find in CDr/y on DD, CDs/y significantly fail to increase complex I activity in the liver and muscle in response to DD. Moreover, in the pancreas of CDs fed DD, complex I activity drops to levels comparable to basal levels in CDr/y provided RD. These findings suggest that, when exposed to DD, the liver, muscle and pancreas of CDs/y with the *Ndufa4* mutation are unable to achieve the high level of mitochondrial activity that is normally required in response to increased demand, most likely because of the absence of the intact NDUFA4 protein.

While investigating complex IV, we found similar activity in CDr/y and CDs/y provided RD. When the animals are fed DD, however, we find a reduction in complex IV activity, similarly in both strains. These findings suggest that the reduction in complex IV activity is primarily related to the DD and not to strain differences, and therefore not to the *Ndufa4* mutation or to the occurrence of diabetes, which develops in CDs/y but not in CDr/y. A possible explanation for the reduction in the activity of complex IV might be related to the low copper content in the DD, copper being an essential co-factor for normal complex IV activity (Horn and Barrientos, 2008).

To complement the study of mitochondrial function, we studied ATP levels, one of the major end products generated by mitochondria. We examined ATP levels in the liver, muscle, kidney and pancreas of animals in the basal nondiabetic state when they were provided normal diet, and when they were challenged with a metabolic load imposed by the DD. We found that ATP levels increase or tend to increase when CDr/y are provided DD compared with RD in the metabolically active organs – liver, muscle and pancreas – but not in the kidneys. We interpret these findings as reflecting increased mitochondrial activity in response to the metabolic load imposed by the DD. In CDs/y fed DD, however, ATP levels do not increase upon demand, and even tend to be lower than those in animals provided RD, suggesting that the *Ndufa4* mutation and the absence of the NDUFA4 protein prevent the mitochondrial ATP response to the diabetogenic challenge that is normally expected when the protein is expressed, as is the case in CDr/y.

As one of the important functions of complex I is regulation of mitochondrial-induced oxidative stress (Sharma et al., 2009), we investigated whether the reduced complex I activity we observed in CDs/y fed DD is associated with increased oxidative stress, an important and uncontested culprit in the pathophysiology of diabetes (Kwak et al., 2010; Lowell and Shulman, 2005; Ma et al., 2012). The increase in serum MDA levels we observed in both CDr/y and CDs/y when fed DD compared with RD indeed suggests enhanced oxidative stress that is induced by exposure to this type of diet. It is notable, however, that the increment in MDA levels is in fact significantly higher in CDs/y on DD than in CDr/y on a similar diet, suggesting that the absence of the NDUFA4 protein and enhanced oxidative stress are associated with the development of diabetes.

The anticipated response to an increase in oxidative stress is activation of antioxidant defenses. The increase in SOD1 and SOD2 levels in the pancreas that we observed in CDr/y fed DD compared with those fed RD is indicative of an appropriate response in this strain to oxidative stress generated by the diet. The lack of such response in the pancreas of CDs/y on DD, on the other hand, suggests that this strain is unable to recruit the necessary defense mechanisms during the increased oxidative stress that is induced by the DD in the presence of the mitochondrial respiratory chain dysfunction that is caused by the absence of the NDUFA4 protein. The CDs/y strain that is fed DD and phenotypically develops diabetes generates, therefore, a higher level of oxidative stress that is poorly dealt with by the antioxidative defenses – in the face of inadequate disposal of reactive oxygen species (ROS) by detoxifying enzymes such as SOD1 and SOD2, and the imbalance between production and detoxification that determines the intracellular ROS concentration (Hasnain et al., 2016) – leading to development of diabetes.

How do these findings further our understanding of the pathophysiology of diabetes in the Cohen diabetic rat model of diet-induced diabetes? Key to integrating all our findings is the realization that there are at least two central players that induce diabetes in our model of complex disease: one is the DD, which constitutes a high metabolic load that is deficient in copper, and the second is the *Ndufa4* mutation. When CDs/y, which carry the *Ndufa4* mutation, function in a normal environment and are fed RD, they are able to maintain complex I activity to a level that is sufficient not to develop diabetes. When fed DD with low copper content, however, CDs/y that carry the *Ndufa4* mutation demonstrate mitochondrial dysfunction, as evidenced by a lack of increase in complex I activity and a reduction in ATP generation in response to the dietary load, leading to enhanced oxidative stress with an impaired response of antioxidant enzymes. Although the exact sequence of events remains unclear, the mitochondria in CDs/y are most likely affected through multiple pathways. Complex I is presented with lack of the NDUFA4 protein in its transmembrane domain, which adversely affects mitochondrial complex I function in the face of increased energetic demand imposed by the DD. Impairment in complex I function owing to the absence of the NDUFA4 protein induces extensive free radical generation, which needs to be effectively dealt with. The important enzymes that hydrolyze these radicals are superoxide dismutases (Cu-Zn-Sod) or SODs, in which copper (Cu) and zinc (Zn) fulfill a major role; in the absence of copper, these enzymes are unable to respond appropriately to the increased level of oxidative stress in CDs/y. The reduced copper in DD is disadvantageous, but not sufficiently – by itself – to prevent the rise in SOD levels, given that CDr/y fed the same copper-poor diet respond adequately with a rise in SOD levels. An alternative explanation for the lack of response of CDs/y, which we tested after 1 month of the DD, at which time CDs had already developed the full-blown diabetic phenotype, is that the persistent enhanced oxidative stress in the absence of a constant supply of copper in diet exhausts, over time, the ability of the pancreas to respond. We conclude that the development of diabetes during exposure to DD in CDs/y is induced by a multiple-pathway hit, as most complex traits are. The *Ndufa4* mutation adversely affects complex I function, leading to increased oxidative stress that is further aggravated by the inability of CDs/y to generate an adequate antioxidative response with SOD1 and SOD2. Additionally, the low copper content in diet adversely affects complex IV function. The end result is a reduction in ATP levels in CDs/y. Together, these multiple hits adversely affect the function of the pancreas and impair insulin release, leading to the development of diabetes. Finally, to the best of our knowledge, our findings consist of the first demonstration of a direct link between a mutation in a gene that is associated with mitochondrial function and the pathophysiology of diet-induced diabetes.

## MATERIALS AND METHODS

### Animals

We obtained the animals from the colony inbred at the Israeli Rat Genome Center (IRGC) of the Barzilai University Medical Center in Ashkelon, Israel (www.irgc.co.il) (Weksler-Zangen et al., 2001; Yagil et al., 1996). We housed the animals in accordance with institutional and governmental regulations, principles of laboratory animal care (NIH publication no. 85-23, revised 1985) and guidelines of the American Society of Physiology for the Care and Use of Laboratory Animals. We maintained climate-controlled conditions and light adjusted to regular timed diurnal cycles. The study protocol was reviewed and approved by the Barzilai University Medical Center Institutional Animal Care and Use Committee (Animal Experimentation Committee).

To elicit the diabetic phenotype in CDs/y, we fed the animals after weaning with standard rat chow and tap water *ad libitum* until age 6-7 weeks. We then switched to DD and copper-poor water *ad libitum*. We custom prepared DD, as previously described (Weksler-Zangen et al., 2001). After 4 weeks on DD, the diabetic phenotype, which consists of an exaggerated hyperglycemic response to glucose loading with impaired insulin response, invariably develops in CDs/y but not in CDr/y (Weksler-Zangen et al., 2001; Yagil et al., 2007).

### Sequencing of the *Ndufa4* gene

We initially sequenced the gene transcript, followed by whole-genome sequencing.

#### Exon sequencing

We surgically dissected out the entire pancreas in anesthetized animals, snap froze the organ and extracted RNA by homogenizing the tissue in Tri Reagent (Molecular Research Center, Cincinnati, OH, USA). To derive cDNA, we used 1 μg total RNA to create the first strand with the Reverse-IT kit (ABgene), applying the random decamer option. We amplified the exon transcripts with custom-designed primers (http://bioinfo.ut.ee/primer3/) from the 5′ to the 3′ untranslated sequence, 5′-TGGCTGTGCTGGAACTCACTCTGT-3′ and 5′-AGACCTGAGGTTTTCCATCTC-3′, and sequenced the PCR products.

Because we found a difference in the size of the PCR products between CDs/y and CDr/y, we tested whether our findings were unique to the pancreas as an organ system, and to the Cohen diabetic rat as a model organism. We therefore derived cDNA from the heart, liver, kidney and muscle of CDs/y and of other nondiabetic rat strains, including SBH/y, SBN/y and SHR rats (Friedman et al., 2003; Yagil et al., 1995; Yamori and Lovenberg, 1987), amplified the transcripts and ran the PCR products in these samples as well.

To sequence the exons, we excised the PCR products by taking 50 μl from the agarose gel, cleaned it with NucleoSpin Extract II and sequenced it using an ABI sequencer at the Crown Human Genome Center of the Weizmann Institute of Science, Rehovot, Israel.

#### Genomic sequencing

We subsequently sequenced the entire gene, including its introns, exons and the promoter region 70 bp upstream to the genes and ∼300 bp in the 3′ transcribed but untranslated region. We extracted genomic DNA by salt precipitation, phenol-chloroform cleaning and amplification by PCR reaction in small 300-700 bp PCR products. We cleaned the PCR products using a mini prep kit and ran them on low-melting-point agarose gel. Using Primer 3 software (http://bioinfo.ut.ee/primer3/), we designed primers designed to cover exons 1-4 and introns 1-3. The PCR reaction consisted of 1 min denaturing at 98°C, 1 min annealing at 53-58°C, and 72°C extension for 1 min×35 cycles. For large PCR products of DNA (1000-4000 bp), we used Phusion High-Fidelity DNA polymerase (Thermo Scientific), with 2 min denaturing at 98°C, annealing for 30 s and 51-56°C extension for 3 min×35. We sequenced the PCR products using an ABI sequencer (Crown Human Genome Center, Weizmann Institute of Science). We used the NCBI-Blast software (http://www.ncbi.nlm.nih.gov/BLAST/) to identify sequence variations between CDs/y and CDr/y. To identify promoter regions motifs, we used the web-based database http://www.cbrc.jp/htbin/nphtf-search.

### Expression of the NDUFA4 protein

We studied the expression of NDUFA4 protein in the liver, muscle, kidney and pancreas of CDs/y and CDr/y. NDUFA4 is a small-sized (∼9KD) membranous-bound protein.

We surgically extracted the organs in the anesthetized animal and froze them at −80°C until processed further. As NDUFA4 resides in the mitochondria, we aimed to enrich the cell membrane fraction. We washed 50-100 μg frozen tissue and minced in PBS-PI (protease inhibitor cocktail from Sigma-Aldrich), dounce homogenized on ice and centrifuged the homogenate at 4000 rpm (1500 ***g***), obtaining a membrane-rich pellet and the supernatant consisting of the cytosolic fraction. We lysed the content in RIPA buffer and used the Bradford method to quantitate the amount of protein extracted.

To extract the protein in the pancreas, we used either the entire organ or obtained islet-enriched homogenates as described by Carter et al. (2009). For the latter, we anesthetized the animals, exsanguinated them, ligated the common bile duct proximal and distal to the pancreatic duct, cannulated the isolated bile duct segment with PE-50 and perfused the pancreas through the pancreatic duct with ∼5 ml of 0.2% collagenase type 4 (Worthington). We then surgically excised the pancreas, inserted the organ into a tube containing collagenase and incubated at 37°C for 11 min with gentle shaking. We diluted the individual samples with ice-cold HBSS buffer and spun for 2 min at 1300 rpm. We re-suspended the pellet in HBSS, layered the suspension on sieve size 40 gauge and centrifuged the resulting sieve for 2 min at 1400 rpm (200 ***g***). We separated the islets by gradient of a mixture of Sigma-Aldrich Histopaque 1119 and 1077, washed twice with HBSS, centrifuged at 3000 rpm (800 ***g***) for 5 min and moved the samples to 1.5 ml tubes containing PBS with proteinase inhibitors (Sigma-Aldrich). We homogenized the islet-enriched solution and froze the homogenates at −80°C until further analysis.

To demonstrate protein expression in the processed tissues, we warmed 50-70 μg protein to 95°C and loaded on Any kD denatured gels (Bio-Rad), ran the gels and transferred to polyvinylidene fluoride (PVDF) membrane. As antibody against NDUFA4, we used ab133698 (epitope 38-81) (Abcam) diluted 1:500-1:1000. The antibody targets mainly exons 3 and 4 and recognizes the protein at ∼10 kD. After blocking the membrane with 5% milk in PBST (0.1%) for 1 h, we incubated with the primary anti-NDUFA4 antibody for 1 h at room temperature or overnight at 4°C. We then washed the membrane with PBST 0.1% Tween and incubated for 1 h with the secondary antibody, goat anti rabbit (1:10,000; Jackson ImmunoResearch). We developed the membranes with Clarity Western ECL Substrate (Bio-Rad).

### NDUFA4 localization within the mitochondrial complexes

This protein has previously been reported to be incorporated as a protein subunit in complex I of the mitochondrial respiratory chain (Garbian et al., 2010; Hayashi et al., 2009; Mishmar et al., 2006). Subsequent studies, however, have pointed to the possibility that NDUFA4 might localize to complex IV (Balsa et al., 2012; Pitceathly et al., 2013; Sinkler et al., 2017). It became thus incumbent upon us to first sort out these conflicting reports and re-explore the localization of the NDUFA4 protein within the mitochondrial complexes in our model. To achieve this goal, we utilized 1D (*n*=3) and 2D (*n*=5) blue native gels, onto which we loaded 60-90 μg protein samples extracted from the liver and kidney of CDr/y with Native Sample Loading Buffer (Bio-Rad) and 5% Coomassie Blue G-250 (Bio-Rad). We ran the native gels for 4 h in parallel with a Native Marker Unstained protein standard. In the 2D gels, we cut the protein lane consisting of complex aggregates, incubated it for 30 min in dissociation buffer (Tris HCl buffer containing 1% sodium dodecyl sulfate and 1% mercaptoethanol) and positioned it on top of the denaturing gel composed of 4% stacking acrylamide/bis gel and 10% acrylamide/bis resolving gel. Both 1D and 2D gel products were transferred to PVDF membranes.

#### Effect of the *Ndufa4* mutation on mitochondrial integrity

We initially tested whether, in CDs/y, the absence of expression of NDUFA4, a structural component of complex I and possibly IV, affects the relative abundance of the mitochondrial complexes when compared with CDr/y, using the OXPHOS blue native western blot antibody cocktail (Abcam, ab110412).

### Effect of the NDUFA4 on mitochondrial function

#### Function of complexes I and IV

In preparation for studying mitochondrial function, we surgically retrieved tissue from the liver, muscle, kidney, spleen, lungs, salivary glands and pancreas of anesthetized CDs/y and CDr/y provided RD or DD and snap froze them until use. We obtained membranous mitochondrial-enriched fractions from 100 mg aliquots of frozen tissue by gentle homogenization (10-30 strokes) in PBS on ice and centrifuging at 13,000 rpm (15,000 ***g***) for 10 min at 4°C. The pellet, consisting of the membranous-rich fraction, was further processed by adding 1× RIPA, homogenization on ice, and centrifugation at 13,000 rpm (15,000 ***g***) for 15 min at 4°C. The supernatant was assayed for complex I and IV activity.

To study complex I activity, we used the Complex I Enzyme Activity Microplate Assay Kit (Abcam, ab109721) that determines OXPHOS mitochondrial complex I (NADH dehydrogenase) activity, as determined by measuring oxidation of NADH to NAD+. To study complex IV activity, we used the Complex 4 Rodent Enzyme Activity Assay Kit (Abcam, ab109911), which determines mitochondrial cytochrome c oxidase enzyme activity. In both assays, absorbance was measured colorimetrically in duplicate or triplicate.

When studying complex I and IV activities, we set the level of activity in CDr/y fed RD as a reference standard for normal activity and compared the results in the other groups with the reference values.

#### ATP levels

We studied ATP levels in CDs/y and CDr/y, representing the end product of the mitochondrial respiratory chain, using the ATP Determination Kit (Life Technologies), a bioluminescence assay for quantitation of ATP using firefly luciferase and its substrate D-luciferin. We homogenized liver tissues with PBS-PI and lysed the cells using a nondenaturing low-detergent buffer. After centrifugation, we measured ATP emission using a multi-detection microplate reader (BioTek, FLx800) and normalized the data for protein concentration.

### Oxidative stress

Oxidative stress is a well-recognized pathophysiological mechanism in the development of diabetes (Hasnain et al., 2016) that has been associated with mitochondrial dysfunction. We studied the level of surrogates of oxidative stress in serum and in tissues of CDs/y and CDr/y provided RD and DD.

#### MDA

We measured lipid peroxidation in serum, an indicator of oxidative stress, using MDA, a naturally occurring product of lipid peroxidation and a TBARS (TCA method) Colorimetric Assay Kit (Cayman Chemical). We expressed the data as percentage of MDA levels found in the reference nondiabetic CDr/y strain provided RD (CDr/y-RD).

#### SOD1, SOD2

As tissue indicators of oxidative stress, we measured the levels of the enzymes SOD1 and SOD2 in the pancreas. We extracted 50-80 μg protein from each organ (as described above), ran the extracts on Mini-Protean TGX Stain-Free Any kD precast gels (Bio-Rad), transferred them to PVDF membranes and blotted with the respective antibodies. For SOD1, we used ab13498 (1:4000; Abcam) (18 kDa), for SOD2 ab13534 (1:4000; Abcam) (26 kDa). We normalized the results with beta-actin as a housekeeping protein, using the anti-beta actin antibody ab227387 (1:20,000; Abcam). We used densitometry with a GelAnalyzer 2010a (GelAnalyzer.jar) to quantitate the results which we expressed as a percentage of the average values found in CDr/y-RD.

### Statistics

Data are shown in the graphs as mean±s.e.m. Between-group analyses were carried out by one-way analysis of variance (ANOVA), and within-group post hoc analysis was by least significance difference (LSD) testing, using Statistica software (www.statsoft.com).
